# Child health care nurses' cultural competence in health visits with children of foreign background

**DOI:** 10.1002/nop2.1393

**Published:** 2022-09-30

**Authors:** Marie Golsäter, Maria Karlsson Fiallos, Sølvi Olsson Vestvik, Hilda Anefur, Maria Harder

**Affiliations:** ^1^ CHILD‐Research Group, School of Health and Welfare Jönköping University Jönköping Sweden; ^2^ Child Health Services, Region Jönköping County Jönköping Sweden; ^3^ Pediatric Outpatient Clinic Region Jönköping County Jönköping Sweden; ^4^ ChiP‐Research Group, School of Health, Care and Social Welfare Mälardalen University Västerås Sweden

**Keywords:** child health care, cultural competence, deductive content analysis, nurses, questionnaire

## Abstract

**Aim:**

The aim of this study is to investigate child health care nurses' cultural competence in health visits with children and their families of foreign background.

**Design:**

A cross‐sectional design combined with a qualitative explorative design.

**Methods:**

The nurses assessed their cultural competence using a modified version of the Clinical Cultural Competency Questionnaire. Interviews were used to obtain a detailed description of the nurses' cultural competence.

**Results:**

The nurses assessed themselves as rather culturally competent. They scored above mid‐score in the total score for cultural competence and on all subscales. Education in cultural diversity at the nurses' workplace had the highest association to cultural competence. The nurses described their awareness as recognizing each child and her/his family rather than their cultural background, and viewing the child as a unique part of her/his cultural context. Despite their high scores on cultural competence, the nurses described a lack of cultural knowledge and explained their need of further knowledge.

## 1 INTRODUCTION

This study focuses on child health care (CHC) nurses' cultural competence when encountering children and their families of foreign background; i.e., children born abroad, or whose parent(s) were born abroad. The significance of the study is related to the fact that children of foreign background constitute a vulnerable group in society at risk of poor health (Carrasco‐Sanz et al., 2018; Hjern, 2012; Kadir et al., 2019).

Children of foreign background more often live in unsafe neighbourhoods and in families supported by social allowance and report poorer health than children of Swedish background (Hjern, 2012). Their health, living conditions and lifestyle habits are influenced by their parents' integration into society (Hjern, 2012). The Swedish CHC services have the prerequisites to promote health among children of foreign background, as they serve all children (0–6 years of age) and their families (Socialstyrelsen, 2014). The CHC services offer free‐of‐charge health visits at well‐baby clinics, based on national guidelines, to all children from birth to 5 years of age in order to promote children's health and development. These visits include measurement of height, weight, vision, hearing and language development, and dialogues on health and lifestyle (Socialstyrelsen, 2014). CHC nurses need to combine their responsibility for health promotion with that for encountering the individual child's needs and must have knowledge about cultural diversity in order to support the health and development of these children at both the individual and group levels. This knowledge is also necessary for contributing to the families' access to the welfare system (Hjern, 2012). Children and their families of foreign background may not understand or may not be supported by, health advice based on Swedish norms and culture if it cannot be transferred to a counterpart in their own culture. Access to the Swedish welfare system is important for children's development and health and makes them less vulnerable (Hjern, 2012).

### 1.1 Background

Previous research involving children and families of foreign background in the CHC services addresses encounters between nurses and parents (Berlin et al., 2006, 2008; Berlin, Nilsson, et al., 2010; Berlin, Tornkvist, et al., 2010). This research shows that when nurses and parents do not share ethnicity, culture or language, the health work becomes complex (Berlin et al., 2006, 2008; Berlin, Nilsson, et al., 2010; Berlin, Tornkvist, et al., 2010; Englund & Rydström, 2012). Belintxon et al. (2020) describes a lack of deeper relationship that enable the parents to receive tailored advice based on their children's needs, as the nurses are unable to capture the parents' descriptions. This complexity makes nurses concerned that they may overlook health risks among the children as they describe a lack of cultural competence. They manage such concerns by scheduling frequent visits with families or by waiting and “hoping for the best” (Berlin, Tornkvist, et al., 2010).

**FIGURE 1:**
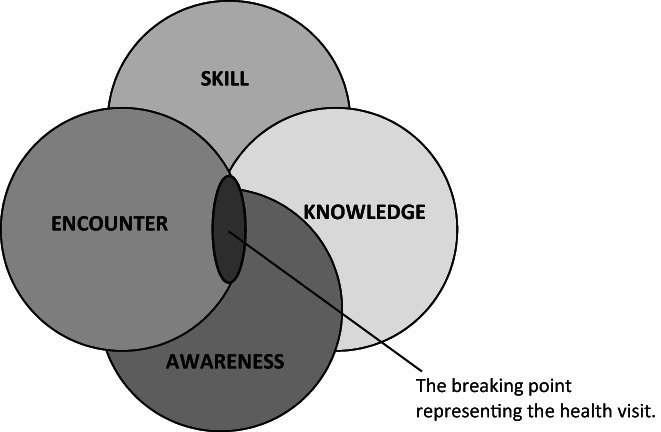
The components in the model of Cultural Competence in the Delivery of Health Care Service and its breaking point representing the health visit where the nurse encounter the child of foreign background

The present study used a theoretical framework based on the Process of Cultural Competence in the Delivery of Healthcare Services model (Campinha‐Bacote, 2002). This model assumes cultural competence to be a process for achieving health care in the patient's cultural context and involves the components of knowledge, skills, encounters and awareness, and how they are expressed in encounters with children of foreign background. The model also requires that nurses continuously develop their cultural competence rather than seeing themselves as already culturally competent. At the breaking point where the components are merged, the health visit with the child and family of foreign background takes part (Figure 1). Health visits with children of foreign background are complex situations, as several circumstances interact and the children's background and culture influence their health and development. Therefore, cultural competence implies understanding culturally based needs and an ability to ask questions to reach an understanding of the cultural diversity. As there is a knowledge gap regarding the encounters in these complex situations involving nurses' cultural competence, there is a need of further investigation.

### 1.2 Aim

The aim of this study was to investigate CHC nurses' cultural competence in health visits with children and their families of foreign background.

Research questions (RQ):

- RQ 1. How do CHC nurses assess their cultural competence in health visits with children and their families of foreign background?
- RQ 2. How does CHC nurses' self‐assessed cultural competence in health visits with children and their families of foreign background correlate to the demographic variables of age, clinical experience, spoken languages, frequency of encountering children of foreign background and level of education in cultural diversity?
- RQ 3. How do CHC nurses describe their cultural competence in health visits with children and their families of foreign background?

## 2 METHOD

### 2.1 Design

This study have a cross‐sectional design to investigate CHC nurses' self‐assessed cultural competence (RQ 1–2) and a qualitative explorative design to obtain detailed descriptions of the CHC nurses' cultural competence (RQ 3). The qualitative part of the study was following the COREQ checklist.

### 2.2 The questionnaire: Participants and procedure

The CHC nurses assessed their cultural competence using a modified version of the Clinical Cultural Competency Questionnaire (CCCTQ) in which questions relevant to the Swedish CHC setting were selected (Wahlström et al., 2020). The modified questionnaire contained 29 questions about cultural competence: knowledge, skills, comfort in encounters and cultural awareness. These areas are based on Campinha‐Bacotes' (Campinha‐Bacote, 2002) model of cultural competence (Table 1). Furthermore, the questionnaire contains demographic variables and questions about education in cultural diversity. The questions regarding cultural competence and education in cultural diversity were assessed on a five‐point Likert scale from very low to very high.

**TABLE 1:** Examples of items in the subscales

Subscale	Examples of items
Cultural knowledge	How knowledgeable are you about the health risks experienced by diverse ethnic groups?
Cultural skills	How skilled are you at conducting a culturally sensitive health visit?
Comfort level in cultural encounters	How comfortable do you feel in encounters with children with limited language proficiency in Swedish?
Cultural awareness	How aware are you of your own prejudices and cultural bias?

The adjusted questionnaire was tested for reliability and internal consistency and showed a Cronbach's alpha score of α.958 for the cultural competence scale and from α.72 to α.95 for the subscales (Wahlström et al., 2020).

At the time for the study, there were about 2,500 CHC nurses working in the 21 counties of Sweden, and each county had a main child healthcare unit (MCHCU). To obtain contact details for the CHC nurses, each MCHCU was contacted and either provided e‐mail addresses for the CHC nurses for distribution of the web questionnaire or chose to distribute an open link to the web questionnaire themselves.

**FIGURE 2:**
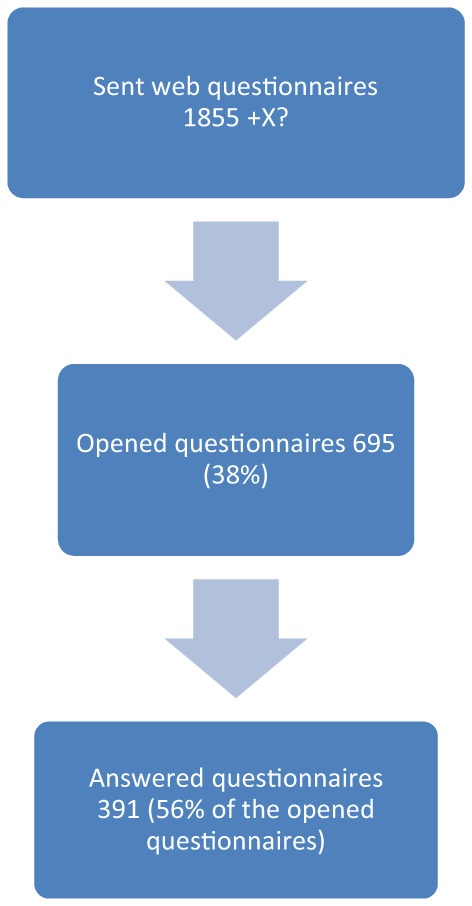
Study sample

The research group sent 1,855 questionnaires to CHC nurses; two reminders were sent to non‐responders. The exact number of questionnaires sent as an open link is unknown. Thus, the total number of eligible participants is unknown (Figure 2).

The participating CHC nurses (*N* = 391) were mostly female (*n* = 390), most were born in Sweden (95.1%), and all had a specialist nursing education, including training in child health nursing. About half of the CHC nurses encountered children of foreign background once or several times a week or more often (Table 2). Besides Swedish, 89.5% of the CHC nurses spoke one or more additional language, typically English.

**TABLE 2:** Characteristics of respondent child health care nurses

Characteristic	*n*	(%)
Gender (*n* = 391)		
Female	390	99.7
Male	1	0.3
Age (*n* = 386); Mean = 47 (*SD*: 9.5)		
≤40 years	111	28.4
41–50 years	128	32.7
51–60 years	104	26.6
≥61 years	46	11.8
Clinical experience in CHC (*n* = 391; Mean = 9.26 (*SD*: 8.8))		
0–5 years	144	36.8
6–10 years	104	26.6
11–15 years	57	14.6
16 < years	86	22.0
Country of origin (*n* = 391)		
Sweden	373	95.1
Nordic country (Sweden excluded)	10	2.6
European country (Nordic countries excluded)	5	1.3
Country outside Europe	3	0.8
Spoken languages (*n* = 391)		
Swedish	40	10.2
One additional language	220	56.0
Two or more additional languages	131	33.5
Specialist education (*n* = 391)		
Public health nursing^a^	269	68.8
Paediatric nursing^a^	92	23.5
Public health and Paediatric nursing	26	6.6
Not specified	4	1.0
Frequency of encountering children of foreign origin (*n* = 391)		
Once or several times a day	66	16.9
Once or several times a week	134	34.3
Once or several times a month	180	46.0
Less than once a month	11	2.8

^a^ Education including training in child healthcare nursing.

### 2.3 The interviews: Participants and procedure

Twenty‐six CHC nurses in one county in south‐eastern Sweden were invited by telephone to participate in the interviews, and 20 accepted. The interviewed CHC nurses worked at 10 different CHC centres and encountered children and families of foreign background every day or several days a week. Furthermore, the CHC nurses were 32–64 years of age and their clinical experience as CHC nurses varied from two to 35 years.

The interview guide was based on the same questions as those in the questionnaire. Each answer was followed up with exploring questions. The content in the interview guide was discussed with a CHC nurse and then pilot‐tested in one interview before the data collection began. The interviewers were three master students supervised by the first author. The interviews were conducted at the CHC centres and lasted 20 to 54 minutes. Each interview was transcribed verbatim.

### 2.4 Analysis

Descriptive statistics were used to describe the study population and the response distributions in the different scales. Spearman correlation coefficient was used to investigate the correlation between demographic variables and the scales of cultural competence. The statistical analyses were performed using IBM SPSS version 24.

**FIGURE 3:**
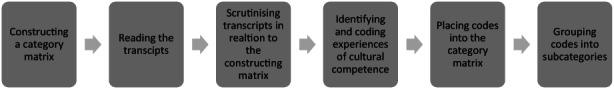
Outline of the analysis process

The interviews were analysed using deductive content analysis (Elo & Kyngas, 2008) based on Campinha‐Bacote's model of cultural competence by the first and the last author trained in qualitative analysis (Figure 3). A category matrix (Table 3) was constructed based on the model. The 20 rich transcripts were read in their entirety and were scrutinized in relation to the model in an ongoing reflection process. The CHC nurses' experiences of their cultural competence were identified and coded, and the codes were placed in the category matrix. Thereafter, the codes in each category were grouped into subcategories according to similarities and differences.

**TABLE 3:** Category matrix with definitions of the core components

Categories	Cultural knowledge	Cultural skills	Cultural encounters	Cultural awareness
Definition	Knowledge about diverse cultural health‐related beliefs, values and incidence of diseases	Clinical ability to gather cultural data, assess a person's issues and offer culturally adjusted care	Comfortable interacting with persons from culturally diverse backgrounds and able to recognize their linguistic needs	Recognition of one's own cultural identity and prejudices to avoid cultural imposition
Subcategories	Gained cultural knowledge theoretically through education	Ability to culturally adjust health care	Comfortable framing a culturally adjusted health visit	Recognizing one's own cultural identity
	Gained cultural knowledge through experiences in clinical practice	Ability to assess children's health and development considering their foreign background	Comfortable interacting with children and their families from culturally diverse backgrounds	Recognizing the cultural influence in the encounter
		Ability to explore a family's knowledge and health conceptions	Comfortable exploring cultural health beliefs	

### 2.5 Ethical considerations

An information letter describing the aim of the study and its procedure was included in the e‐mails with the web questionnaire. The part of the study related to the questionnaire was approved by the Regional Ethical Committee in Uppsala, Sweden (DNR: 2016/560). The interviews were performed according to ethical principles (Association, 2009). Before the interviews, participants received both oral and written information about the study, confidentiality, and how the data would be handled and were told they could withdraw their participation at any time.

## 3 RESULTS

The results are presented as quantitative measures based on the questionnaire and qualitative descriptions from the interviews.

### 3.1 Cultural competence

The nurses assess themselves as rather culturally competent. They score above mid‐score in the total score for cultural competence and on all four subscales (Table 4). There is an association between the degree of education in cultural diversity and each subscale. Education in cultural diversity at the nurses' workplace has the highest association with the different subscales, and the total scale of cultural competence (Table 5). There was a minor correlation between years of work experience and spoken language in some of the subscales, and in the total scale (Table 5).

**TABLE 4:** Child healthcare nurses' cultural competence, cultural knowledge, cultural skills, comfort level in encounters and cultural awareness, and education in cultural diversity

*n* = 391	Maximum score	Midscore^a^	Mean (*SD*)	Median
Cultural competence	145	87	99.57 (15.17)	99.58
Cultural knowledge	30	18	19.00 (4.36)	19.00
Cultural skills	45	27	29.19 (5.10)	29.00
Cultural comfort level in encounters	40	24	26.43 (5.10)	26.00
Cultural awareness	30	18	24.95 (3.65)	25.00

^a^ Midscore = all responses are at midpoint on the rating scale (3 = neither high nor low level of …).

**TABLE 5:** Correlations between demographic variables and cultural competence and subscales

	Cultural competence and subscales				
	Cultural competence	Knowledge	Skills	Comfort in encounters	Awareness
Age	.099	.108*	.107*	.056	.046
Years of work experience	.147**	.119*	.163**	.105*	.073
Language	.167**	.187*	.88	.130*	.186**
Frequency of encountering children of foreign origin	.090	.057	.089	.069	.068
Education in cultural diversity (ECD)	.418**	.380**	.370**	.365**	.170**
ECD in nursing education	.180**	.195**	.168**	.143**	.021
ECD in specialist nursing education	.265**	.234**	.262**	.214**	.096
ECD at work	.401**	.364**	.349**	.370**	.162**

* Correlation is significant at the .05 level.

** Correlation is significant at the .01 level.

In the following, the nurses' cultural competence is described from its categories and their associated subcategories (Table 3).

### 3.2 Cultural knowledge

The nurses' process of developing knowledge about diverse cultural health‐related beliefs, values and incidence of diseases in various ethnic groups is described as *gained theoretically by education* or *gained by experience in clinical practice*. This process entails both searching for knowledge and putting effort into maintaining it.

Despite the high scores on cultural competence (Table 4), the nurses describe a lack of *cultural knowledge gained theoretically by education* regarding cultural diversity in both the nursing education and the nursing specialist education. This is confirmed by the nurses' reports of little to no education in cultural diversity in their nursing education (91.6%) and in their nursing specialist education (86.4%) (Table 4). The nurses describe the theoretically gained knowledge as being arranged by the MCHCU as lectures. Still, most of the nurses (80%) report receiving little to no education in the workplace (Table 2). The nurses say they need knowledge in cultural diversity in order to understand a family's cultural context and be able to support them and also because the population is becoming more diverse. Still, they emphasize the importance of keeping the individual family in focus in order to avoid theories on cultural diversity becoming preconceptions. The need of additional theoretical cultural knowledge is displayed in the following quotation:

There's a lot more and it isn't immigration; they're refugees, and what they've been through and what experiences they carry with them varies. We would absolutely like more knowledge about that. (I1)

Further, theoretical knowledge refers to the national guidelines regarding the programme for refugees, each child's vaccination status and circumcision traditions. The nurses describe an uncertainty regarding how cultural aspects are discussed in the national guidelines and in the programme. In their estimations, almost half of the nurses assess their knowledge about cultural aspects as neither low nor high for national guidelines (43%) and cultural aspects in the programme (42.2%).

The *cultural knowledge gained by experiences in clinical practice* concerns the nurses' knowledge about the ethnic groups they encounter, and these groups various cultural characteristics, health risks and health differences. They describe themselves as having good knowledge about the various ethnic groups at their CHC centre, which is reflected in estimations of having rather or very high knowledge (56.2%).

The nurses describe that knowledge about cultural characteristics as symbols, e.g., clothes, food and traditions, is gained by encountering the families several times. The nurses primarily estimate their knowledge about cultural characteristics as neither high nor low (43.5%). One nurse describes her knowledge about cultural characteristics as follows:

There are lots of symbols, such as wearing special clothes, special jewellery. You learn over time what they mean. Having a dot on your forehead or a special hat worn in a special way, wearing a sari in a Muslim or Hindu way, wearing Romani clothing and understanding what it means, knowing about how a woman covers herself, being Sikh and wearing a turban, having various orthodox characteristics. (I4)

Regarding knowledge concerning health risks and health differences among ethnic groups, the nurses describe uncertainty. Mentioned health risks include smoking and mental illness. While the nurses estimate their knowledge of health risks as neither high nor low (38.3%), nonetheless 34.3% assess their knowledge as rather high. The same pattern is shown regarding the nurses' estimations of their knowledge about health differences. These are described as the families' lack of knowledge regarding dental health and child security relating to level of education, socioeconomic background and the family's refugee status.

### 3.3 Cultural skills

The nurses' cultural skills involve their ability to gather cultural data and assess a child's or family's issues considering their foreign background and offer culturally adjusted care. Cultural skills comprise *the ability to culturally adjust the health care, the ability to assess children*'*s health and development considering their foreign background*, and *the ability to explore the family*'*s knowledge and health conceptions*.

Regarding *the ability to culturally adjust the health care*, the nurses describe using a pliable approach towards the child and family's needs, with respect for their cultural background:

I approach my families in the same way: you have respect for how close you can get and how intrusive you can be. (I8)

One way of acting pliable is explaining the meaning of the health visit in a simplified way to ensure comprehension. Still, the nurses describe uncertainty as to the extent to which a health visit needs adjustment to a family's culture. This is shown in their assessments: only 4.6% assess themselves as very qualified and 26.9% as rather qualified in conducting culturally adjusted health visits. Still, the nurses describe themselves as relatively qualified in greeting children individually. Regarding how to greet parents, to some extent the nurses describe an uncertainty in how to act as when the father does not want to shake hands.

The nurses describe their *ability to assess children*'*s physical health and development considering their foreign background* that may differ among children. Concerning assessing the child's development, especially language, the nurses describe uncertainty. This is verified in their estimations, with only 5.6% assessing themselves as very qualified and 39.1% as being rather qualified to assess children's health and development. In order to ensure their health assessments, the family is invited to frequent visits to the CHC centre.

The nurses describe their *ability to explore the family*'*s knowledge and health conceptions* as being able to listen, being curious and asking questions. This approach is important for showing respect for the families' fears and concerns and may reduce linguistic and cultural misunderstandings. Still, the parents and children's ability to express themselves and the nurses' ability to understand them influence the situation. While the nurses describe this as a challenge, more than half of the nurses assess themselves as very or rather qualified in exploring families' knowledge and conceptions about health:

It's often the case with many parents that they want to talk with us about what they've done, if they've had something. Then you find out what they've done and you consider whether it's unhealthy. (I4)

### 3.4 Cultural encounters

Taking part in cultural encounters can be described as the CHC nurses' interactions with families of culturally diverse backgrounds in order to culturally adjust the health care and recognize linguistic needs.

Cultural encounters include: *being comfortable framing a culturally adjusted health visit, being comfortable interacting with children and their families from culturally diverse backgrounds*, and *being comfortable exploring cultural health beliefs*.

*Being comfortable framing a culturally adjusted health visit* involves the nurses designing health visits in the same way as with families of Swedish background, but they prepare additionally by ordering an interpreter and allocating more time. Specific procedures used with children and families of foreign background are described as using body language and drawings, in addition to verbal communication and information material in different languages:

But we have these pictures, which is really good, for both Swedish children and non‐Swedish children … and … where you go through and tell what it is we're going to do, and what's going to happen, and show in the pictures and explain. (I5)

The nurses foremost assess themselves as rather or highly comfortable (51.4%) in encounters with children with limited knowledge in Swedish.

*Being comfortable interacting with children and their families from culturally diverse backgrounds* entails the need to build trust by having knowledge, listening, showing a positive attitude and trying to capture the child's interest. Further, the nurses focus on the unique family, independent of their background. They feel certain in interacting with children of foreign background, as children are always children regardless of cultural background. Almost three‐quarters of the nurses assessed themselves as being very or rather certain in encounters with children of different cultural backgrounds. Further, they describe feeling certain because they have the national guidelines to rely on when monitoring the child's health and development. This is reflected in their estimations and in the following:

I try to respond to the family with kindness and curiosity, and I think I get pretty far with that way. Then there's certainly a lot I miss. (I3)

The nurses describe how the CHC services are experienced positively among families, even if aspects of the health visit may be incomprehensible to them. They mention the importance of recognizing different cultural modes of expressing, e.g., pain and suffering. Half of the nurses assessed themselves as uncertain in understanding the meaning of special cultural gestures.

*Being comfortable exploring cultural beliefs* is necessary, and the nurses describe that they ask questions in this endeavour. This helps them understand the family and to see their own cultural beliefs from a different perspective. If there are culturally based actions in a family that affect the child in a negative way, the nurses describe how they try to talk to the parents in a humble way to avoid offending them and that they can usually find a solution together:

I have to start with the problem and try to understand and base it on what's causing the problem and try to explain what I'm thinking, but it's difficult. I believe people want to do the best for their child, and then it ends up not that great; it's hard. (I3)

About 40% of the nurses assessed themselves as being very certain or rather certain in providing advice on how cultural practices and habits can affect health, and the same amount assessed themselves as neither certain nor uncertain (39.4%).

### 3.5 Cultural awareness

The nurses score highest on cultural awareness (Table 4) and describe their experiences of it as *recognizing one*'*s own cultural identity* and *recognizing the cultural influence in the encounter*. These subcategories are intertwined, as the nurses' recognition of their own cultural identity is a prerequisite for not allowing their cultural preconceptions to influence the encounter. Further, it is in the encounter that they discover their cultural awareness.

The nurses describe that encounters with persons from various cultures contribute to their *recognition of their own cultural identity*. Half of the nurses assess themselves as rather aware of their own ethnic or cultural identity, and one‐fourth as very aware. Their recognition builds on reflections regarded as necessary in order to be able to understand various cultures and not miss any problems a family may mention. The nurses reflect on their cultural inheritance, religion, generational belonging and upbringing, as they believe these aspects affect their values and preconceptions.

Further, close connections with cultural diversities are described as a strength, as the knowledge gained from these experiences helps in understanding families of foreign background. Still, the nurses' point out that, whatever the background, it is important to encounter families with humility:

I still think that … that we always still have to see each encounter as unique, and that it's a unique individual, a unique family we have in front of us … and that one should be curious about that instead of thinking*aha here comes a Muslim family, I know exactly how it's going to be. (I10)

The importance of recognizing the unique child and family lies in the nurses' knowledge that in a specific ethnic group there are cultural similarities and differences in the same way as there are between different ethnic groups. These descriptions can be related to the fact that 58.8% of the nurses state that it is important that healthcare professionals have the opportunity to receive training in and information about cultural diversity.

The nurses describe that they need to *recognize cultural influences in the encounter* in order to encounter the family with openness and in a culturally adjusted way, to avoid misunderstandings and create trust. In interaction with children, the nurses foremost assess cultural awareness as rather important (48.1%) and in interaction with parents foremost as very important (51.4%). They also describe how other nurses can influence the encounter, which may relate to their estimates of cultural awareness towards professional colleagues as rather or very important (46%, 34%). Further, their professional culture, the workplace culture and the overall culture in society are described as influences in the encounter. The following quote shows the complexity of cultural influences in the encounter:

I live in a Swedish culture, but I also come from my own family culture. That can also have an affect … and society's culture, and the fact that I'm a nurse perhaps also means that I'm a certain way … actually you're quite conscious of it. Above all, you're aware of your culture when you encounter others from other countries. (I6)

However, the nurses state that it is primarily the child's personality, rather than their culture, that influences the encounter.

## 4 DISCUSSION

The results show that the nurses assess themselves as rather culturally competent and that education in cultural diversity at the nurses' workplace had the highest association to cultural competence. Further, that nurses score highest on cultural awareness.

The nurses in the present study assess themselves as rather culturally competent. However, the components in Campinha‐Bacotes's model do not exist individually. They rather constitute parts of each other (Campinha‐Bacote, 2002) and the nurses' descriptions show the connection between cultural awareness and cultural knowledge. Both are gained in the cultural encounters with the child and her/his family of foreign background. Still, there is no correlation between the nurses' frequencies of encountering children and their families of foreign background and their awareness, knowledge, skills or comfort in the encounters. In contrast to the present study, a study exploring cultural competences among school nurses showed that their cultural competence was related to the frequencies at which they encountered children and their families of foreign background (Wahlström et al., 2020). However, the school nurses indicated that they encountered children of foreign background more often compared with the CHC nurses, which might explain the differences. Another study focusing on nursing students found that an environment with diverse population and taking care of patients of diverse cultures are predictors of cultural competence (Cruz et al., 2016).

Cultural competence in Campinha‐Bacotes's model (Campinha‐Bacote, 2002) is described as achieving health care in the cultural context of the patient. The nurses in the present study score highest on cultural awareness which they describe as recognizing each child and her/his family and not only their culture and foreign background. Such an approach to children and their families of foreign background in encounters shows the CHC nurses' clinical practice as individual support, with the child's individual needs prioritized over health issues at the group level (Olander, 2003). On the one hand, CHC nurses need to recognize each child and her/his family as part of a child‐centred care (Coyne et al., 2018). But on the other hand, they also have an obligation to the child and family as a possible part of a vulnerable population in society at risk of poor health (Carrasco‐Sanz et al., 2018). The nurses need to consider their health promotion assignment as a whole, i.e., their dual assignment (Coyne et al., 2018; Olander, 2003) to work with health promotion at a group level based on standardized assessments and to simultaneously encounter the individual child's needs. It seems as the assignment regarding health for the population is neglected which may be due to their cultural knowledge. The nurses assess their cultural knowledge as neither high nor low, and they point out that they lack and need further cultural knowledge. They receive little to no theoretical knowledge in their education and they have mostly gained knowledge about cultural diversity through experience. Mareno and Hart (2014) show the similarly regarding nurses' description of lack of knowledge and education in cultural diversity. The lack of theoretical knowledge regarding cultural diversity may be a result of having received one's nursing education or specialist nursing education a long time ago, when the immigrated population was small. Cicolini et al. (2015) argue that nurses need to be better prepared to encounter various health needs as diversity in population grows. The lack of knowledge can also be understood as a continuous need of replenishment and opportunity for reflection to maintain and develop cultural competence, as this is an ongoing process (Alizadeh & Chavan, 2016; Campinha‐Bacote, 2002). The desire of nurses to develop their cultural competence is evident in the present study. This desire to strengthen their competence is similarly described by Eche and Aronowitz (2017) regarding nurses in paediatric oncology.

However, the individual support approach is equally important as a population support approach. Research conducted in hospitals from the view of parents of foreign background shows that nurses' engagement, respect, sensitivity and understanding in the encounters constitute the foundation of a functional encounter between nurses and parents of different cultural backgrounds. More important than knowledge about families' culture, traditions and religion are the nurses' skills at being active listeners, open‐minded and honest and pliable (Tavallali et al., 2017). In the present study, the nurses describe their cultural skills as listening, showing a positive attitude and focusing on the unique family to build a trustful relationship and acting pliable. Belintxon et al. (2020) verify the need of a relationship that enable nurses to capture parents' descriptions and tailor the care for each individual family. Further Karim et al. (2020) highlight that a thoughtful relationship can help to alleviate the uncertainty that parents describe in the encounter with child health care that is different from that in the home country. In the light of these experiences from parents, the CHC nurses' clinical practice in the present study, based on an individual support approach, can be understood as a way to compensate for their uncertainty regarding their cultural knowledge regarding health issues on population level. Education in cultural diversity at the nurses' workplace had the highest association to cultural competence. Still, the nurses' report receiving little to no education in cultural competence in the workplace. Further, the nurses describe a lack of cultural aspects in the national programme and guidelines, i.e., the Swedish national child health programme (Socialstyrelsen, 2014). Such lack of cultural aspects may contribute to the nurses working more from an individual support approach. The child health programme does not guide them when assessing the children's health and therefore the nurses are omitted to shape the health visits as best as they can. In the study, the nurses specific describe their uncertainty about their skills in assessing language development and the lack of guidelines and tools for such assessments in children of foreign background which also is described in earlier studies (Nayeb et al., 2015). The nurses' actions to compensate for their uncertainty is to offer frequent visits (Berlin et al., 2008). The nurses insecurity may also affect the parents and earlier studies have pointed out that when parents of foreign background do not feel secure, their possibilities to express themselves are hindered (Berlin, Tornkvist, & Hylander, 2010) and they seek information and support outside the healthcare system (Karim et al., 2020). Cicolini et al. (2015) suggests that training in and provision of culturally competent care among other things improve communication, satisfaction with the care provided and health status. But also an appropriate utilization of the health care system.

The present study indicates the need to develop an educational intervention in the national MCHCU to ensure evidence‐based care for children and their families of foreign background. In such an intervention, the nurses' experiences need to be reflected from a theoretical perspective to further develop their knowledge, skills and awareness in the complex encounters with children and their families (Harder et al., 2018). Campinha‐Bacote' s model of cultural competence (Campinha‐Bacote, 2002) could be used as a foundation for the intervention, as it requires nurses to develop cultural competence rather than seeing themselves as already culturally competent.

### 4.1 Limitations

In interpreting the results from this study, some limitations must be considered. Regarding the questionnaire, some of the MCHCUs chose to distribute an open link to the web questionnaire themselves; thus we cannot be certain that all nurses had access to the web questionnaire. The response rate was low, with 391 responders out of a total of about 2,500 CHC nurses working in Sweden. However, for the 695 opened questionnaires the response rate was 56% and nurses from all 21 counties in Sweden were represented. Further, there was variation in the answers both in assessing their cultural competence and in their frequency of encountering children of foreign background. This indicates that it was not only CHC nurses with a special interest in children of foreign background who participated.

The interview participants were CHC nurses from one county, which could be interpreted as a limitation. However, these nurses worked at ten different CHC centres and among them there was variation in work experience and the frequency of encountering children of foreign background.

There were some discrepancies between the data gathered from the questionnaire and the interviews, which could be understood as a limitation in understanding the questions in the questionnaire or answering with no further reflection. Through the interviews based on the same model as the questionnaire, however, a more in‐depth description of the nurses' reflections on their cultural competence emerged, which could be seen as strengthening the answers from the questionnaires.

## 5 CONCLUSION

This study shows that the participating CHC nurses lack theoretical knowledge of cultural diversities. Despite the high scores on cultural competence, the interviewed nurses describe a lack of knowledge regarding cultural diversity, and cite a need of further knowledge as the population is becoming more culturally diverse.

### 5.1 Relevance for clinical practice

Children of foreign background constitute a vulnerable group in society at risk of poor health (Carrasco‐Sanz et al., 2018; Hjern, 2012; Kadir et al., 2019). Therefore, CHC nurses need continuous opportunity to reflect on cultural encounters to maintain and develop cultural competence as part of promoting health among children of foreign background and ensure evidence‐based care. Educational interventions regarding cultural diversity are necessary to provide each child with the health care they need. But also, to ensure equal access to the Swedish welfare system (Hjern, 2012).

## AUTHOR CONTRIBUTIONS

Study design: Marie Golsäter and Maria Harder. Data collection: Maria Karlsson Fiallos, Sölvi Olsson Vestvik, Hilda Anefur. Data analysis: Marie Golsäter and Maria Harder. Manuscript writing Marie Golsäter, Maria Karlsson Fiallos, Sölvi Olsson Vestvik, Hilda Anefur and Maria Harder.

All authors have agreed on the final version and meet at least one of the following criteria [recommended bythe ICMJE (http://www.icmje.org/recommendations/)]:

• substantial contributions to conception and design, acquisition of data or analysis and interpretation of data;

• drafting the article or revising it critically for important intellectual content.

## FUNDING INFORMATION

The authors received founding from Futurum, Region Jönköping, Sweden.

## CONFLICT OF INTEREST

No conflict of interest has been declared by the authors.

## Data Availability

Data available on request due to privacy/ethical restrictions.

## References

[nop21393-bib-0001] Alizadeh, S. , & Chavan, M. (2016). Cultural competence dimensions and outcomes: A systematic review of the literature. Health & Social Care in the Community, 24(6), e117–e130. 10.1111/hsc.12293 26499469

[nop21393-bib-0002] Association, W. M. (2009). Declaration of Helsinki. Ethical principles for medical research involving human subjects. Jahrbuch für Wissenschaft Und Ethik, 14(1), 233–238.

[nop21393-bib-0003] Belintxon, M. , Dogra, N. , McGee, P. , Pumar‐Mendez, M. J. , & Lopez‐Dicastillo, O. (2020). Encounters between children's nurses and culturally diverse parents in primary health care. Nursing & Health Sciences, 22(2), 273–282. 10.1111/nhs.12683 31943713

[nop21393-bib-0004] Berlin, A. , Hylander, I. , & Tornkvist, L. (2008). Primary child health care nurses' assessment of health risks in children of foreign origin and their parents—A theoretical model. Scandinavien Journal of Caring Sciences, 22(1), 118–127. 10.1111/j.1471-6712.2007.00533.x 18269431

[nop21393-bib-0005] Berlin, A. , Johansson, S.‐E. , & Tornkvist, L. (2006). Working conditions and cultural competence when interacting with children and parents of foreign origin—primary child health Nurses' opinions. Scandinavian Journal of Caring Sciences, 20(2), 160–168. 16756521 10.1111/j.1471-6712.2006.00393.x

[nop21393-bib-0006] Berlin, A. , Nilsson, G. , & Törnkvist, L. (2010). Cultural competence among Swedish child health nurses after specific training: A randomized trial. Nursing & Health Sciences, 12(3), 381–391. 10.1111/j.1442-2018.2010.00542.x 20727091

[nop21393-bib-0007] Berlin, A. , Tornkvist, L. , & Hylander, I. (2010). Watchfully checking rapport with the primary child health care nurses—A theoretical model from the perspective of parents of foreign origin. BMC Nursing, 9. 10.1186/1472-6955-9-14 PMC2918611 20646287

[nop21393-bib-0008] Campinha‐Bacote, J. (2002). The process of cultural competence in the delivery of healthcare services: A model of care. Journal of Transcultural Nursing, 13(3), 181–184. 10.1177/10459602013003003 12113146

[nop21393-bib-0009] Carrasco‐Sanz, A. , Leiva‐Gea, I. , Martin‐Alvarez, L. , del Torso, S. , van Esso, D. , Hadjipanayis, A. , Kadir, A. , Ruiz‐Canela, J. , Perez‐Gonzalez, O. , & Grossman, Z. (2018). Migrant children's health problems, care needs, and inequalities: European primary care paediatricians' perspective. Child: Care, Health and Development, 44(2), 183–187. 10.1111/cch.12538 29159977

[nop21393-bib-0010] Cicolini, G. , Della Pelle, C. , Comparcini, D. , Tomietto, M. , Cerratti, F. , Schim, S. M. , Di Giovanni, P. , & Simonetti, V. (2015). Cultural competence among Italian nurses: A multicentric survey. Journal of Nursing Scholarship, 47(6), 536–543. 10.1111/jnu.12165 26444447

[nop21393-bib-0011] Coyne, I. , Holmström, I. , & Söderbäck, M. (2018). Centeredness in healthcare: A concept synthesis of family‐centered care, person‐centered care and child‐centered care. Journal of Pediatric Nursing, 42, 45–56. 10.1016/j.pedn.2018.07.001 30219299

[nop21393-bib-0012] Cruz, J. P. , Estacio, J. C. , Bagtang, C. E. , & Colet, P. C. (2016). Predictors of cultural competence among nursing students in The Philippines: A cross‐sectional study. Nurse Education Today, 46, 121–126. 10.1016/j.nedt.2016.09.001 27636832

[nop21393-bib-0013] Eche, I. J. , & Aronowitz, T. (2017). Evaluating cultural competence of pediatric oncology nurses at a teaching hospital: A pilot study. Journal of Pediatric Oncology Nursing, 34(6), 422–426. 10.1177/1043454217713452 28660797

[nop21393-bib-0014] Elo, S. , & Kyngas, H. (2008). The qualitative content analysis process. Journal of Advanced Nursing, 62(1), 107–115. 18352969 10.1111/j.1365-2648.2007.04569.x

[nop21393-bib-0015] Englund, A.‐C. D. , & Rydström, I. (2012). “I have to turn myself inside out”: Caring for immigrant families of children with asthma. Clinical Nursing Research, 21(2), 224–242. 10.1177/1054773812438915 22473272

[nop21393-bib-0016] Harder, M. , Söderbäck, M. , & Ranheim, A. (2018). Health care professionals' perspective on children's participation in health care situations: Encounters in mutuality and alienation. International Journal of Qualitative Studies on Health and Well‐Being, 13(1), 1555421. 10.1080/17482631.2018.1555421 30704374 PMC6319471

[nop21393-bib-0017] Hjern, A. (2012). Migration and public health:Health in Sweden: The National Public Health Report 2012. Chapter 13. Scandinavian Journal of Public Health, 40(9_suppl), 255–267. 10.1177/1403494812459610 23238411

[nop21393-bib-0018] Kadir, A. , Battersby, A. , Spencer, N. , & Hjern, A. (2019). Children on the move in Europe: A narrative review of the evidence on the health risks, health needs and health policy for asylum seeking, refugee and undocumented children. BMJ Paediatrics Open, 3(1), bmjpo‐2018‐000364. 10.1136/bmjpo-2018-000364 PMC6361329 30815582

[nop21393-bib-0019] Karim, N. , Boyle, B. , Lohan, M. , & Kerr, C. (2020). Immigrant parents' experiences of accessing child healthcare services in a host country: A qualitative thematic synthesis. Journal of Advanced Nursing, 76(7), 1509–1519. 10.1111/jan.14358 32189345

[nop21393-bib-0020] Mareno, N. , & Hart, P. L. (2014). Cultural competency among nurses with undergraduate and graduate degrees: Implications for nursing education. Nursing Education Perspectives, 35(2), 83–88. 10.5480/12-834.1 24783722

[nop21393-bib-0021] Nayeb, L. , Wallby, T. , Westerlund, M. , Salameh, E.‐K. , & Sarkadi, A. (2015). Child healthcare nurses believe that bilingual children show slower language development, simplify screening procedures and delay referrals. Acta Paediatrica, 104(2), 198–205. 10.1111/apa.12834 25327143

[nop21393-bib-0022] Olander, E. (2003). Hälsovägledning i barnhälsovården, syntetiseringav två uppdrag [health counseling in child health care, synthesizing two missions in child healthcare]. Doctoral thesis. Malmö University.

[nop21393-bib-0023] Socialstyrelsen . (2014). Vägledning för barnhälsovården[Guidance for child health care].

[nop21393-bib-0024] Tavallali, A. G. , Jirwe, M. , & Kabir, Z. N. (2017). Cross‐cultural care encounters in paediatric care: Minority ethnic parents' experiences. Scandinavian Journal of Caring Sciences, 31(1), 54–62. 10.1111/scs.12314 26800093

[nop21393-bib-0025] Wahlström, E. , Harder, M. , Granlund, M. , Holmström, I. K. , Larm, P. , & Golsäter, M. (2020). School nurses' self‐assessed cultural competence when encountering children of foreign origin: A cross‐sectional study. Nursing & Health Sciences, 22(2), 226–234. 10.1111/nhs.12663 31729131

